# Supplementary material for: Molecular Epidemiological Survey for Degenerative Myelopathy in German Shepherd Dogs in Japan: Allele Frequency and Clinical Progression Rate
Source: Animals (Basel). 2022 Jun 27;12(13):1647. doi: 10.3390/ani12131647 (PMC9264911; doi:10.3390/ani12131647)
Supplement: Supplementary file 1 [file animals-12-01647-s001.zip › animals-1748451-Table S1.pdf]

Table S1. Number of German Shepherd Dogs (GSDs) registered by the Japan Kennel Club (JKC) and Japan Shepherd Dog Registration Society (JSV).

Year	JKC*			JSV
	Total purebred dogs	GSDs	GSDs (%)	GSDs
1999	424,061	819	0.19	
2000	447,978	865	0.19	
2001	475,603	813	0.17	
2002	523,530	776	0.15	
2003	575,792	843	0.15	
2004	561,713	766	0.14	
2005	554,141	733	0.13	
2006	533,941	564	0.11	
2007	491,429	449	0.09	
2008	465,540	433	0.09	
2009	439,238	361	0.08	
2010	392,958	314	0.08	
2011	366,065	379	0.10	919
2012	351,114	351	0.10	871
2013	326,009	309	0.09	809
2014	306,438	365	0.12	668
2015	301,605	273	0.09	506
2016	300,470	289	0.10	508
2017	295,910	318	0.11	450
2018	292,906	259	0.09	372
2019	293,659	234	0.08	381
2020	305,532	179	0.06	301
2021	320,493	414	0.13	334
AVG	406,353	483	0.11	556

* Data are publicly available at a website of JKC (https://www.jkc.or.jp (Accessed 11 May 2022)). ** Data are provided by JSV.
